# Can Radiological Renal Artery Parameters Predict Acute Kidney Injury in Infective Endocarditis Surgery?—From Imaging to Outcomes

**DOI:** 10.3390/diagnostics14222527

**Published:** 2024-11-12

**Authors:** Christian Dinges, Elke Boxhammer, Iris Kremser, Katja Gansterer, Johannes Steindl, Nikolaos Schörghofer, Christoph Knapitsch, Reinhard Kaufmann, Uta C. Hoppe, Matthias Hammerer, Klaus Hergan, Bernhard Scharinger

**Affiliations:** 1 Department of Cardiovascular and Endovascular Surgery, Paracelsus Medical University of Salzburg, 5020 Salzburg, Austria; 2 Department of Internal Medicine II, Division of Cardiology, Paracelsus Medical University of Salzburg, 5020 Salzburg, Austria; 3 Department of Medical and Chemical Laboratory Diagnostics, Paracelsus Medical University of Salzburg, 5020 Salzburg, Austria; 4 Department of Radiology, Paracelsus Medical University of Salzburg, 5020 Salzburg, Austria

**Keywords:** acute kidney injury, computed tomography, endocarditis, renal artery anatomy, renal artery calcifications, valve surgery

## Abstract

**Background:** Infective endocarditis (IE) poses significant challenges in cardiovascular medicine, often necessitating valvular surgery to manage severe complications. Postoperative acute kidney injury (AKI) is a notable complication affecting patient outcomes. While clinical and procedural factors have been well studied, the role of radiological renal artery parameters in AKI risk remains underexplored. **Methods:** This retrospective study analyzed 80 patients with IE who underwent valvular surgery from 2013 to 2021, focusing on postoperative AKI as defined by the Kidney Disease: Improving Global Outcomes (KDIGO) criteria. Radiological parameters, including renal artery calcification, renal ostial calcification, the presence of renal infarctions, and additional arteries, were assessed using preoperative computed tomography (CT). Statistical analyses included binary logistic and linear regression models, Kaplan–Meier survival curves, and Cox proportional hazard regression to explore associations between these parameters and AKI incidence, creatinine levels, and mortality. **Results:** Out of 80 patients, 31 (38.8%) developed AKI. No significant differences were found in baseline characteristics or radiological parameters between the AKI+ and AKI− groups. Binary logistic and linear regression analyses revealed no substantial relationship between anatomical factors and AKI risk or creatinine levels. However, Cox regression identified “additional renal artery” as a significant predictor of 1-month mortality (HR: 1.747, 95% CI: 1.024–2.979, *p* = 0.041) but not for 6- or 12-month mortality. **Conclusions:** Radiological anatomical factors, including calcifications and additional arteries, did not significantly impact AKI risk in IE patients undergoing valvular surgery. However, the presence of additional arteries was associated with increased short-term mortality. These findings suggest the need for further research to elucidate factors contributing to AKI and mortality in this context.

## 1. Introduction

Infective endocarditis (IE) represents a significant challenge within cardiovascular medicine, characterized by the invasion of pathogens into the bloodstream, which leads to severe and often life-threatening complications [1,2]. The disease spectrum ranges from mild, asymptomatic cases to severe infections with profound systemic effects. Effective management typically necessitates prompt and aggressive treatment, often including surgical intervention [3,4,5].

Valvular surgery is a cornerstone in treating of IE, particularly when the infection is complicated by uncontrollable infection, embolic events, or severe valvular dysfunction. This surgical approach aims to eradicate the infectious focus, repair or replace damaged heart valves, and restore cardiac function [3,6,7]. Despite its critical role in managing the acute phase of IE, the postoperative period presents its own set of challenges with acute kidney injury (AKI) emerging as a significant complication [8]. AKI is a sudden decline in kidney function that can severely impact patient outcomes both in short term and long term [8,9,10]. The kidneys’ high metabolic demands and complex vascular network make them particularly vulnerable to the hemodynamic fluctuations and inflammatory responses associated with cardiac surgery [11]. Although advancements in perioperative care have improved the management of IE and its complications, AKI remains a prevalent concern, contributing to increased morbidity, mortality, and prolonged hospitalizations [12]. The relationship between IE, valvular surgery, and AKI is multifaceted and warrants further exploration. Understanding the factors that contribute to AKI in this context is crucial for developing targeted interventions aimed at reducing its incidence and mitigating its impact. Traditionally, risk assessments for AKI have focused on clinical and procedural factors [13]; however, there is growing interest in how radiological anatomical parameters might influence the risk of AKI.

Radiological imaging provides detailed insights into anatomical features that may impact renal function, such as the calcification of renal arteries or ostial regions, the presence of renal infarctions, and additional arterial structures [14]. These anatomical parameters could potentially affect renal perfusion and susceptibility to AKI in the setting of valvular surgery for IE. Despite their potential significance, these factors are often overlooked in standard risk assessments. This study aims to address this gap by integrating radiological anatomical parameters into the analysis of AKI risk among patients with IE undergoing valvular surgery. By examining how factors like renal artery calcification, ostial calcification, and the presence of additional arteries relate to the occurrence and severity of AKI, this research seeks to enhance our understanding of how these variables contribute to postoperative renal complications.

## 2. Materials and Methods

### 2.1. Study Population

This study initially involved the enrollment of 130 patients diagnosed with infective endocarditis (IE) at a major tertiary care center in Salzburg, Austria. All participants were scheduled for valve surgery and were consecutively recruited over a nine-year period from 2013 to 2021. The primary focus of the study was the occurrence of postoperative AKI. To maintain a clear scope, individuals who presented with AKI prior to surgery were excluded. This exclusion extended to those with potential tubular or glomerular damage or those who had been exposed to relevant nephrotoxic antimicrobial agents. Additionally, patients requiring dialysis or hemo(dia)filtration prior to surgery were not included in the study. To ensure a consistent study cohort, only patients with stable renal function and stable renal blood parameters before undergoing surgery were selected. A total of 80 patients had an available CT scan to perform radiological analysis and could be included in the final analysis. All data for this study were analyzed retrospectively.

### 2.2. Ethics Statement

The research protocol was reviewed and approved by the Ethics Commission of the State of Salzburg (EK: 1109/2023). The study was conducted in line with the ethical standards outlined in the Declaration of Helsinki and adhered to Good Clinical Practice guidelines. Given the retrospective nature of the study, the requirement for patient consent was waived by the Ethics Commission.

### 2.3. Infective Endocarditis Criteria

All patients included in the study met the Duke criteria for the diagnosis of IE or the criteria specified in the 2015 European Society of Cardiology (ESC) guidelines [15], which were applicable at the time the surgical intervention was indicated.

### 2.4. Indication for Valve Surgery

The decision to proceed with surgical intervention on the affected valve(s) due to IE was made by a multidisciplinary endocarditis team [15,16]. This team included cardiologists, cardiac surgeons, anesthetists, and infectious disease specialists. Surgical treatment was pursued when one or more of the following conditions were present: heart failure or severe valve dysfunction secondary to IE, uncontrolled infection, or a high risk of embolization.

### 2.5. Valve Replacement Surgery for Endocarditis

For patients requiring valve replacement due to endocarditis, the surgical process started under general anesthesia, which was followed by a median sternotomy to provide access to the heart. To sustain systemic circulation during the procedure, cardiopulmonary bypass was initiated, and the heart was temporarily stopped using cardioplegia to protect the myocardial tissue. The aorta was clamped to separate the heart from the systemic circulation. The diseased valve was carefully removed with extensive debridement of all infected and dead tissue to reduce the likelihood of recurrent infection. Depending on the patient’s specific anatomy and condition, a prosthetic valve or homograft was implanted. The surgical area was thoroughly examined to ensure the complete removal of the infection before closing the chest. After surgery, patients were closely observed for any complications, and antimicrobial treatment was continued to ensure the infection was fully eradicated.

### 2.6. Acute Kidney Injury

The objective of this study was to evaluate the incidence of postoperative AKI in patients with IE by monitoring creatinine levels pre- and post-surgery. The incidence of AKI was determined using the Kidney Disease: Improving Global Outcomes (KDIGO) criteria. Since continuous and complete urine output data were not available in all patient records, the serum creatinine-based criteria were utilized to define and classify AKI in this cohort in a follow-up period up to 7 days post-surgery.

### 2.7. Computed Tomography Protocol

All included study patients routinely underwent a non-contrast CT of the head and a contrast-enhanced CT of the thorax, abdomen, and pelvis with intravenous contrast media during the arterial and portal venous phases to assess, among others, embolic infarcts and calcified as well as non-calcified plaques in the ascending aorta preoperatively. CT scans were performed using multidetector CT scanners, specifically second-generation, multidetector 256- or 128-slice dual-source CT scanners (Revolution, General Electric Healthcare, Chicago, IL, USA or Somatom Definition AS+, Siemens Healthcare, Erlangen, Germany) with tube voltage adjusted according to patient size (80–120 kVp) and active tube current modulation. A bolus-tracking technique was employed, administering a 100 mL bolus of non-ionic iodinated contrast media followed by 70 mL of saline solution at a flow rate of 3.5–5 mL/s.

### 2.8. Radiological Analysis and Measurements

**Figure 1:**
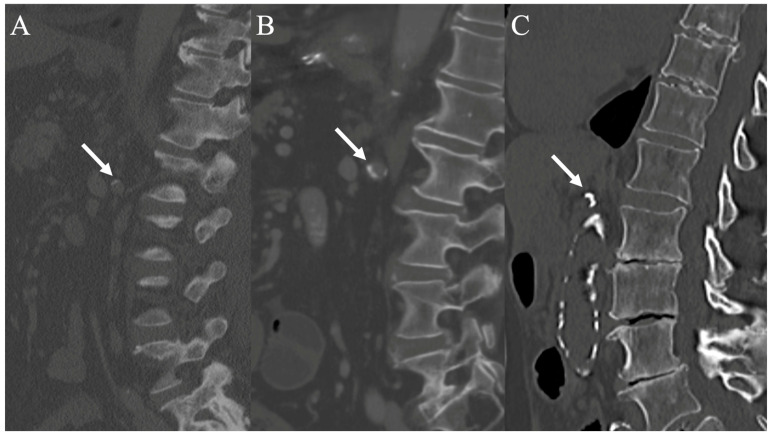
CT scans show the renal artery ostium region in the sagittal plane to assess the morphology and circumference of calcifications. (**A**) shows thin linear (eggshell-type) calcifications (score 1), (**B**) shows thick linear calcifications (score 2), and (**C**) shows bulky calcifications (score 3) (white arrows).

Image analysis and measurements were performed by a board-certified radiologist subspecialized in cardiovascular imaging using a stationary workstation with dedicated software (Deep Unity Diagnostics 1.1.1.1, Dedalus Healthcare, Milan, Italy). To assess renal infarcts, CT images were analyzed in three planes (axial, coronal, and sagittal) during both the corticomedullary and nephrogenic phases. Renal infarcts were defined as focal, wedge-shaped parenchymal defects involving both the cortex and medulla with results recorded as either “yes” or “no”. Additional renal arteries were assessed in the arterial phase with results recorded as either “yes” or “no”. Renal artery calcifications were evaluated based on the scoring system for iliac arteries by Davis et al. [17]. Calcifications were assessed in the left and right ostial regions and the left and right main renal arteries during the arterial phase, using a standard bone window, and in three planes with a 3 mm slice thickness. A semiquantitative scoring system, including the morphology and circumference of involvement for the ostial region and the morphology, circumference, and length of involvement for the artery, was applied to evaluate the degree and distribution of calcifications (Table 1 and Figure 1). The calcification categories were assigned a numeric score from 0 to 3 for morphology (0 = no calcifications, 1 = thin linear, eggshell-type calcifications under 1 mm, 2 = thick linear calcifications over 1 mm, 3 = bulky calcifications over 2 mm) and 0 to 4 for length and circumference (0 = no calcifications, 1 = 1–25%, 2 = 26–50%, 3 = 51–75%, 4 = 76–100%), ranging from none to worst. The highest score was used when different characteristics were present. Therefore, the ostial score ranges from 0 to 7, and the arterial score ranges from 0 to 11.

### 2.9. Outcomes Investigated

The primary outcomes of this study focused on evaluating the incidence of AKI in patients with IE undergoing valvular surgery. To understand the role of anatomical factors in AKI risk, we analyzed radiological parameters such as renal artery calcification, calcification of the ostium, the presence of renal infarctions, and the number of additional renal arteries. These variables were assessed through preoperative imaging studies, especially CT, to explore their association with AKI incidence and severity. By comprehensively evaluating these outcomes, this study aims to elucidate the relationship between radiological anatomical features and the risk of AKI in IE patients undergoing valvular surgery, ultimately contributing to improved risk assessment and management strategies.

### 2.10. Statistical Analysis

A post hoc power analysis (G*Power 3.1) revealed that the study had a power of 50–60%. The power analysis was conducted with an alpha (α) level of 0.05 (the standard threshold for statistical significance) and a beta (β) level of 0.2 (corresponding to a statistical power of 80%).

Further statistical analyses and graphical representations were performed using SPSS software (Version 25.0, SPSS Inc., Armonk, NY, USA). The Kolmogorov–Smirnov–Lilliefors test was applied to evaluate the normality of the data distribution. Variables that were normally distributed were summarized as mean ± standard deviation (SD) and compared using an unpaired Student’s *t*-test. For variables not following a normal distribution, data were presented as median and interquartile range (IQR), and comparisons were made using the Mann–Whitney U test. Categorical variables were expressed as frequencies and percentages and analyzed using the chi-square test.

To explore differences in survival between patients with and without postoperative AKI (AKI+ vs. AKI−), Kaplan–Meier survival curves were generated, accompanied by log–rank tests and numbers at risk, covering a follow-up period from 30 days to 1 year. A univariate binary logistic regression model was employed to calculate hazard ratios (HRs) and 95% confidence intervals (CIs) for radiological factors associated with AKI. For better comparability, metric variables were standardized using z-transformation. A multivariable binary logistic regression could not be conducted due to lacking significance in univariate analysis. Furthermore, a simple linear regression was calculated to examine a connection between creatinine 1 day preoperatively up to 2 days postoperatively and radiological parameters regarding the aforementioned radiological parameters. At last, a univariate Cox proportional hazard regression model in dependence of different radiological parameters was used to calculate HR and 95% CI associated with short-term survival (up to one year). Again, a z-transformation was performed for metric data for better compatibility. Afterwards, a multivariable Cox regression was performed to assess independent predictors of mortality. Therefore, covariates associated with mortality in the univariate analysis (*p* < 0.100) were entered, and a backward variable elimination was performed. Statistical significance was set at *p* < 0.050 for all analyses.

## 3. Results

### 3.1. Flow Chart of Study Cohort

**Figure 2:**
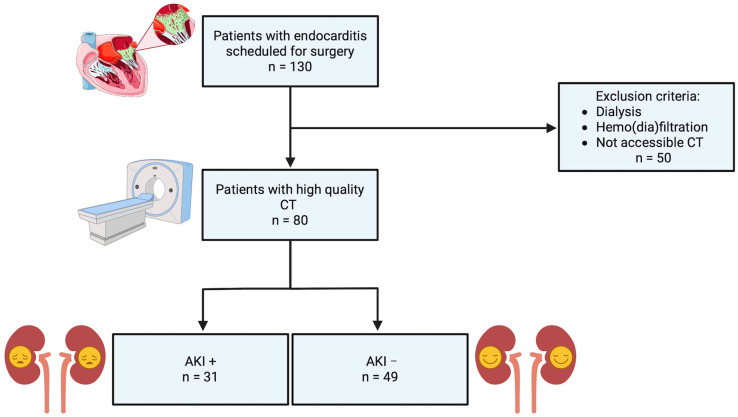
Flow chart of study cohort (created with www.BioRender.com); CT: computed tomography; AKI: acute kidney injury.

The study population initially comprised 130 patients diagnosed with IE and scheduled for valve surgery. As depicted in the flow chart of Figure 2, 50 patients were excluded due to the need for dialysis, hemodiafiltration, or inaccessibility of CT imaging. This exclusion resulted in 80 patients with high-quality CT scans available for analysis. These 80 patients were subsequently categorized into two groups: 31 patients who developed postoperative acute kidney injury (AKI+) and 49 patients who did not experience AKI (AKI−).

### 3.2. Baseline Characteristics

The study included 80 patients with 31 (38.8%) developing acute kidney injury (AKI+). There were no significant differences in gender distribution (72.5% male overall, *p* = 0.807) or pre-existing conditions such as diabetes, hypertension, or cardiovascular disease between the AKI+ and AKI− groups. However, beta-blocker and diuretic use was significantly higher in the AKI+ group (*p* = 0.025 and *p* = 0.015, respectively).

There were no significant differences between the groups regarding preoperative and intraoperative conditions, including surgery urgency and the number of affected native and prosthetic heart valves. Notably, in-hospital mortality was significantly higher in the AKI+ group (32.3% vs. 4.1%, *p* = 0.001). Importantly, baseline creatinine levels did not differ significantly between the AKI+ and AKI− groups before surgery (*p* = 0.207), suggesting that renal function was similar at the study’s outset. A relevant overview of the baseline characteristics is available in Table 2.

### 3.3. Kaplan–Meier Analysis

**Figure 3:**
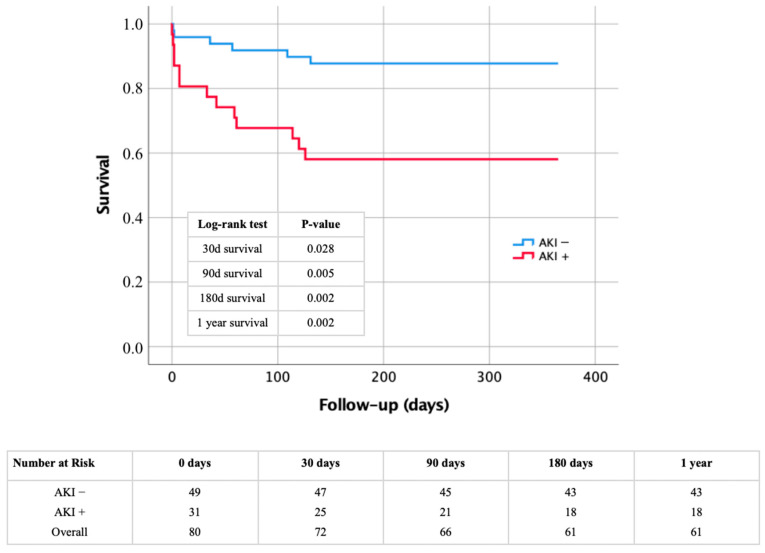
Kaplan–Meier curve with corresponding numbers at risk and log–rank tests for detection of short-term morality in dependence of presence or absence of AKI. AKI: acute kidney injury.

The impact of AKI as a major factor in early deaths was also apparent in short-term results over a 1-year span. This is illustrated by the Kaplan–Meier curve of Figure 3 and confirmed through the log–rank tests and risk data also shown in Figure 3. Patients who developed AKI post-surgery had notably higher mortality rates throughout the follow-up period of 30 days to 1 year. Specifically, the mortality rate for those with AKI at 30 days was 19.4%, increasing to 41.9% after 6 and 12 months.

### 3.4. Comparison of Anatomical Measures in Patients with and Without AKI

In the analysis of calcium load in renal ostia and arteries between patients with AKI and those without (Table 3), we found no significant differences across most of the measured variables. Specifically, measures such as the degree of calcification in the entire ostia and arteries and their combined values did not show notable variation between AKI+ and AKI− groups. The proportion of additional arteries and infarctions also remained similar across the groups. This trend was consistent when examining individual components on the right and left sides, including ostium morphology, circumference, and artery dimensions. Overall, our findings suggest that AKI status does not markedly influence these particular anatomical and pathological measures.

### 3.5. Binary Logistic Regression

The binary logistic regression analysis of Table 4 explored the relationship between various anatomical and clinical factors with the occurrence of AKI. Overall, none of the examined factors showed a significant association with AKI. The analysis of ostium entirety yielded a hazard ratio of 1.232 (95% CI: 0.785–1.932) with a *p*-value of 0.364, suggesting no significant effect. Similarly, artery entirety had a hazard ratio of 1.056 (95% CI: 0.675–1.652) with a *p*-value of 0.812, indicating no substantial relationship with the development of AKI. When combining ostium and artery entirety, the hazard ratio was 1.173 (95% CI: 0.750–1.835), with a *p*-value of 0.485, again showing no significant association. Additional artery involvement and infarction also did not demonstrate significant effects, with hazard ratios of 1.086 (95% CI: 0.694–1.699) and 1.008 (95% CI: 0.642–1.584), respectively, both having non-significant *p*-values.

Separate analyses for the right and left renal arteries provided consistent findings, with none of the factors, including ostium morphology, artery morphology, and their respective circumferences and lengths, showing a significant association with AKI. The *p*-values remained above the significance threshold across all factors, confirming the lack of a substantial relationship between these variables and the risk of AKI.

### 3.6. Linear Logistic Regression

The linear regression analysis (Table 5) examined how various factors influenced creatinine levels at different time points: one day preoperatively (D-1), the day of surgery (D0) and up to four days postoperatively (D1, D2, D3, D4). Although some factors were statistically significant, their practical relevance was limited due to low R-squared values.

For creatinine levels at D-1, anatomical factors like calcification of the ostium and artery had significant *p*-values but low R-squared values (around 0.15 or less), indicating minimal explanatory power. Similarly, at D0, while some associations were statistically significant, the models explained only a small fraction of the variance in creatinine levels with R-squared values generally below 0.06.

At D1 to D4, the models continued to show statistically significant *p*-values for several factors but with even lower R-squared values (below 0.07 or even less).

The R-squared values were generally low across all time points, ranging from 0.003 to 0.152. This indicates that the models only explain a small fraction of the variability in creatinine levels, suggesting that other factors not included in the models may have a significant influence.

### 3.7. Cox Hazard Regression

The Cox hazard regression analysis assessed the impact of various radiological parameters on mortality at 1, 6, and 12 months following valvular surgery as can be observed in Table 6.

For 1-month mortality, the univariate analysis identified two predictors with a *p* ≤ 0.100: “Additional Artery” and “Infarction”. “Additional Artery” had a hazard ratio (HR) of 1.742 (95% CI: 1.038–2.924) with a *p*-value of 0.036, while “Infarction” had an HR of 1.571 (95% CI: 0.934–2.640) with a *p*-value of 0.088. However, in the multivariable analysis, only “Additional Artery” remained a significant predictor, with an HR of 1.747 (95% CI: 1.024–2.979), and a *p*-value of 0.041.

At 6 and 12 months, none of the variables showed significant associations with mortality. Hazard ratios were close to 1 and *p*-values were above 0.15, indicating that these factors did not significantly impact long-term mortality.

## 4. Discussion

### 4.1. Complex Pathophysiology of AKI in Cardiac Surgery

The absence of a significant impact from radiological parameters, such as renal artery calcification and additional renal arteries, on the occurrence of AKI in patients undergoing valvular surgery for IE underscores the complex and multifactorial nature of AKI in this setting. AKI in the context of cardiac surgery is driven by a myriad of factors, many of which may have a more profound influence than anatomical variations [18].

Hemodynamic fluctuations during surgery, including rapid changes in blood pressure and cardiac output, play a crucial role in renal perfusion. The kidneys, with their high metabolic demands and dense vascular network, are particularly susceptible to these fluctuations [19,20]. In this context, it is possible that the systemic hemodynamic changes induced by surgery exert a more significant influence on renal function than localized anatomical features like vascular calcification or anatomical anomalies.

Moreover, the use of nephrotoxic agents during the perioperative period, including certain antibiotics and contrast media, can further compound the risk of AKI. These agents can induce renal tubular damage, adding to the hemodynamic and inflammatory stress on the kidneys [21,22]. As a result, the influence of anatomical factors may be minimized in the context of these potent, multifactorial insults.

### 4.2. Alternative Pathomechanisms: Inflammatory and Immune-Mediated Damage

Beyond anatomical considerations, the pathogenesis of AKI in patients with IE undergoing cardiac surgery is likely driven by inflammatory and immune-mediated mechanisms [23]. IE itself is a highly inflammatory condition that can trigger a systemic inflammatory response syndrome (SIRS). This response, particularly when combined with the inflammatory insult of cardiac surgery, can lead to direct renal injury. The release of pro-inflammatory cytokines, activation of the complement system, and recruitment of immune cells can all contribute to renal endothelial damage, increased vascular permeability, and subsequent renal dysfunction [24,25].

Moreover, patients with IE are at an increased risk of embolic events, including microemboli that can obstruct the microvasculature of the kidneys [26]. These microemboli can cause localized ischemia and infarction, leading to renal injury that is independent of the larger anatomical features assessed by imaging studies [27]. This embolic phenomenon represents a distinct pathomechanism for AKI that is not directly related to the degree of arterial calcification or the presence of additional renal arteries, further explaining the lack of correlation between these radiological parameters and AKI in our findings.

### 4.3. Clinical and Procedural Factors

It is important to consider the impact of clinical and procedural factors on the risk of AKI. The management of patients undergoing valvular surgery for IE involves complex decisions regarding surgical technique, anesthetic management, and perioperative care [28], all of which can significantly influence renal outcomes. For instance, the duration and method of cardiopulmonary bypass, as well as the management of intraoperative fluid balance and blood pressure, are critical determinants of renal perfusion and function during surgery [29]. Even minor variations in these factors can lead to significant differences in the risk of AKI, potentially outweighing the impact of anatomical features.

Additionally, patient-specific factors, such as pre-existing comorbidities like diabetes, hypertension, and chronic kidney disease, are well-established risk factors for AKI. These conditions, especially in combination, may predispose patients to renal dysfunction, making them more susceptible to the effects of surgery and perioperative hemodynamic changes [30]. In this context, the baseline health of the patient, including their renal reserve and overall cardiovascular function, may play a more significant role in determining AKI risk than the radiological parameters evaluated in this study.

### 4.4. Impact of Additional Renal Arteries on Short-Term Mortality

The finding that additional renal arteries are linked to higher 1-month mortality may seem counterintuitive at first, as these arteries could provide redundant blood flow to the kidneys, potentially safeguarding against ischemic injury. However, several factors could explain this association:

- Increased Surgical Complexity and Hemodynamic Instability: The presence of multiple renal arteries introduces anatomical complexity, which can complicate both the surgical procedure and perioperative management. During valvular surgery, maintaining stable renal perfusion is critical, particularly in the context of endocarditis, where systemic inflammation and infection are already taxing the cardiovascular system. The additional renal arteries may alter the expected hemodynamic responses, making it more difficult to manage renal blood flow intraoperatively. This could lead to suboptimal perfusion, acute kidney injury (AKI), or other complications that contribute to higher short-term mortality [31].
- Underlying Systemic Vascular Disease: The presence of additional renal arteries might not be merely an anatomical variation but could also reflect underlying systemic vascular conditions. These additional arteries may be more susceptible to atherosclerosis or other vascular pathologies, potentially compromising renal function. Given the systemic nature of endocarditis, which often involves widespread vascular damage, the presence of additional renal arteries might exacerbate the already increased risk of vascular complications during surgery [32].
- Impact on Renal Function and Perfusion: While additional renal arteries might theoretically provide compensatory blood flow, their presence could also indicate a more complex vascular network that is prone to dysfunction, particularly under the stress of major surgery. For example, if these arteries are narrower or more tortuous than normal, they could contribute to uneven perfusion or be more easily compromised by embolic events during surgery [33,34]. In patients with endocarditis, who are already at risk for embolization due to vegetations on the valves, this could further impair renal perfusion and increase the risk of acute postoperative complications, including mortality.

Interestingly, the analysis showed that the impact of additional renal arteries on mortality did not persist beyond the first month. At 6 and 12 months, hazard ratios were close to 1, and *p*-values were well above the threshold for significance. This suggests that the presence of additional renal arteries may primarily influence immediate postoperative outcomes rather than long-term survival. One possible explanation is that patients who survive the initial postoperative period may experience recovery of renal function or compensatory mechanisms that mitigate the initial impact of additional renal arteries. Moreover, the long-term management of these patients including the optimization of renal and cardiovascular function reduces the influence of anatomical variations on survival.

The association between additional renal arteries and higher short-term mortality underscores the need for careful preoperative assessment and intraoperative management in patients with endocarditis undergoing valve surgery. Identifying patients with complex renal artery anatomy could help clinicians anticipate potential complications and tailor their surgical and anesthetic approaches accordingly. Further research is needed to explore the exact mechanisms by which additional renal arteries influence surgical outcomes and to determine whether specific interventions could mitigate the associated risks.

## 5. Limitations

This study, while providing valuable insights into the association between radiological anatomical parameters and postoperative AKI in patients with IE undergoing valvular surgery, has several limitations that must be acknowledged.

- Retrospective Design: The retrospective nature of this study introduces inherent limitations, including potential selection bias and incomplete data. Although efforts were made to include a well-defined cohort, the reliance on existing records for preoperative and postoperative data may have resulted in missed or inaccurate information. Specifically, the absence of complete urine output data for some patients restricted our ability to utilize all available AKI diagnostic criteria, potentially affecting the accuracy of AKI assessment.
- Selection Bias: Due to the retrospective design and the single-center setting, there is a possibility of selection bias, as patient inclusion was dependent on available clinical and radiological data, which may not be representative of the broader population of IE patients undergoing surgery.
- Sample Size, Generalizability, and Power: The final analysis was limited to 80 patients out of an initial cohort of 130, which is primarily due to exclusions related to missing CT imaging or pre-existing renal conditions. This reduced sample size may affect the generalizability of our findings. Furthermore, the study was conducted at a single tertiary care center, which may limit the applicability of the results to other populations with different demographic or clinical characteristics. Additionally, a post hoc power analysis (G*Power 3.1) revealed that the study had a power of 50–60%, which is lower than the conventional threshold of 80% for detecting significant effects. The power analysis was conducted with an alpha (α) level of 0.05 (the standard threshold for statistical significance) and a beta (β) level of 0.2 (corresponding to a statistical power of 80%). This suggests that the study may have been underpowered to detect subtle relationships between radiological parameters and AKI, and a larger sample size would likely be necessary to improve the robustness of the findings.
- Radiological Parameter Analysis: While we assessed a range of radiological parameters, including renal artery calcification and ostial calcification, the associations with AKI were not significant. It is possible that other unmeasured anatomical or physiological factors might play a role in AKI risk that were not captured by our imaging analyses. Additionally, the quality of CT imaging and the variability in radiological interpretation could impact the accuracy of the anatomical measurements and their associations with AKI.

## 6. Conclusions

The lack of a significant impact of radiological parameters on AKI risk in patients with IE undergoing valvular surgery suggests that these anatomical features may not be the primary drivers of postoperative kidney injury in this population. The multifactorial nature of AKI, combined with potential compensatory mechanisms and the influence of other clinical factors, likely plays a more substantial role. This finding underscores the complexity of AKI pathogenesis in cardiac surgery and suggests that future research should focus on integrating a broader range of clinical, procedural, and potentially molecular factors to better understand and mitigate this serious complication.

## Figures and Tables

**Table 1:** Semiquantitative calcification scoring system for the renal artery and ostium, including morphology, circumference, and length.

Category	Score	Definition
Morphology	0	No calcifications
	1	Thin linear (eggshell-type) calcifications under 1 mm
	2	Thick linear calcifications over 1 mm
	3	Bulky calcifications over 2 mm
Circumference and length (artery only)	0	No calcifications
	1	1–25% involvement
	2	26–50%
	3	51–75%
	4	76–100%

**Table 2:** Baseline characteristics of study cohort.

	Total	AKI+	AKI−	*p*-Value
No. (%)				
Total	80 (100)	31 (38.8)	49 (61.2)	-
Gender (male)	58 (72.5)	22 (71.0)	36 (73.5)	0.807
Pre-existing Conditions				
Diabetes Mellitus	16 (20.0)	4 (12.9)	12 (24.5)	0.207
Arterial Hypertension	41 (51.2)	18 (58.1)	23 (46.9)	0.332
CVD	26 (32.5)	11 (35.5)	15 (30.6)	0.650
Atrial Fibrillation	23 (28.7)	12 (38.7)	11 (22.4)	0.117
COPD	7 (8.8)	5 (16.1)	2 (4.1)	0.063
PAOD	6 (7.5)	3 (9.7)	3 (6.1)	0.556
Chronic Kidney Disease	10 (12.5)	3 (9.7)	7 (14.3)	0.544
Chronic Heart Failure	16 (20.0)	4 (12.9)	12 (24.5)	0.207
Premedication				
Beta-Blocker	39 (48.8)	20 (64.5)	19 (38.8)	0.025
Diuretics	33 (41.3)	18 (58.1)	15 (30.6)	0.015
ACEI/ARB/ARNI	24 (30.0)	13 (41.9)	11 (22.4)	0.064
Preoperative Conditions				
Elective Surgery	3 (3.8)	1 (3.2)	2 (4.1)	0.392
Urgent Surgery	61 (76.3)	24 (77.4)	37 (75.5)	0.775
Emergency Surgery	16 (20.0)	6 (19.4)	10 (20.4)	0.810
Intraoperative Conditions				
Prosthetic Valve Endocarditis	24 (30.0)	12 (38.5)	12 (24.5)	0.176
Endocarditis of One Heart Valve	66 (82.5)	24 (77.4)	42 (85.7)	0.341
Endocarditis of Two Heart Valves	14 (17.5)	7 (22.6)	7 (14.3)	0.341
Endocarditis of Three Heart Valves	0 (0.0)	0 (0.0)	0 (0.0)	1.000
Aortic Valve Endocarditis	54 (67.5)	23 (74.2)	31 (63.3)	0.256
Mitral Valve Endocarditis	36 (45.0)	14 (45.2)	22 (44.9)	0.831
Tricuspid Valve Endocarditis	3 (3.8)	1 (3.2)	2 (4.1)	0.743
Pulmonary Valve Endocarditis	1 (1.3)	0 (0.0)	1 (2.0)	0.798
Postoperative Conditions				
ECMO	4 (5.0)	3 (9.7)	1 (2.0)	0.127
Bleeding/Tamponade	8 (10.0)	5 (16.1)	3 (6.1)	0.146
Stroke	4 (5.0)	2 (6.5)	2 (4.1)	0.636
Valvular Complications	1 (1.3)	1 (3.2)	0 (0.0)	0.206
Third-Degree Atrioventricular Block	8 (10.0)	2 (6.5)	6 (12.2)	0.400
Sepsis	1 (1.3)	0 (0.0)	1 (2.0)	0.423
In-Hospital Death	12 (15.0)	10 (32.3)	2 (4.1)	0.001
Mean ± SD				
Age (years)	61.5 ± 13.9	65.2 ± 11.9	59.2 ± 14.7	0.183
Height (cm)	173.2 ± 8.3	172.2 ± 7.6	173.8 ± 8.8	0.387
Weight (kg)	80.4 ± 15.9	85.2 ± 16.0	77.4 ± 15.2	0.564
BMI (kg/m^2^)	26.8 ± 4.8	28.7 ± 4.9	25.6 ± 4.3	0.822
BSA (m^2^)	1.9 ± 0.2	2.0 ± 0.2	1.9 ± 0.2	0.498
EuroScore II	10.6 ± 10.4	14.1 ± 10.4	8.5 ± 9.9	0.393
Surgery Time (min)	284.7 ± 115.1	335.2 ± 108.3	252.7 ± 108.6	0.280
Clamping Time (min)	110.0 ± 55.6	135.2 ± 63.2	94.1 ± 44.0	0.295
Perfusion Time (min)	168.2 ± 90.0	211.7 ± 103.3	140.7 ± 68.4	0.393
Median ± IQR				
LVEF (%)	55.0 ± 4.5	55.0 ± 4.0	55.0 ± 5.3	0.760
Creatinine D-1	0.9 ± 0.5	0.9 ± 0.4	0.8 ± 0.5	0.207
Creatinine D0	1.3 ± 0.8	1.5 ± 0.2	0.9 ± 0.5	0.083
Creatinine D1	1.4 ± 0.9	1.6 ± 0.4	0.8 ± 0.6	0.015
Creatinine D2	1.4 ± 1.4	2.2 ± 1.0	0.9 ± 0.7	<0.001
Creatinine D3	1.5 ± 1.0	1.8 ± 1.0	1.1 ± 0.7	<0.001
Creatinine D4	1.3 ± 0.9	1.5 ± 1.0	1.0 ± 0.7	<0.001
Creatinine D5	1.2 ± 0.7	1.3 ± 0.9	0.9 ± 0.7	<0.001
Creatinine D6	1.2 ± 0.9	1.4 ± 1.1	1.0 ± 0.7	<0.001
Creatinine D7	1.3 ± 0.8	1.3 ± 1.2	1.0 ± 0.7	0.003
Urine Output 1–2 h postop. (mL)	40.0 ± 57.5	30.0 ± 25.0	65.0 ± 73.8	0.089
Urine Output 2–3 h postop. (mL)	40.0 ± 65.0	20.0 ± 30.0	55.0 ± 75.0	0.123
Fluid Volume—intraop. (L)	3.2 ± 1.7	3.5 ± 1.9	3.2 ± 1.7	0.381

CVD: cardiovascular disease; COPD: chronic obstructive pulmonary disease; PAOD: peripheral arterial occlusive disease; ACEI: angiotensin-converting-enzyme inhibitor; ARB: angiotensin receptor blocker; ARNI: angiotensin receptor–neprilysin inhibitor; ECMO: extracorporeal membrane oxygenation; BMI: body mass index; BSA: body surface area; LVEF: left ventricular ejection fraction;.

**Table 3:** Radiological renal parameters of the overall cohort as well as a comparison of patients with and without AKI.

	Overall	AKI+	AKI−	*p*-Value
Overall				
Ostium Entirety	2.5 ± 6.3	3.0 ± 8.3	0.0 ± 6.3	0.186
Artery Entirety	0.0 ± 0.0	0.0 ± 2.3	0.0 ± 0.0	0.517
Ostium + Artery Entirety	3.0 ± 9.0	3.0 ± 9.0	0.0 ± 9.3	0.154
Additional Artery (%)	21/80	9/31	12/49	0.225
Infarction (%)	18/80	6/31	12/49	0.307
Right				
Ostium Morphology	0.0 ± 2.0	0.0 ± 2.0	0.0 ± 2.0	0.868
Ostium Circumference	0.0 ± 1.0	0.0 ± 1.0	0.0 ± 1.0	0.788
Ostium Entirety	0.0 ± 3.0	0.0 ± 3.0	0.0 ± 3.3	0.702
Artery Morphology	0.0 ± 0.0	0.0 ± 0.0	0.0 ± 0.0	0.737
Artery Circumference	0.0 ± 0.0	0.0 ± 0.0	0.0 ± 0.0	0.866
Artery Length	0.0 ±0.0	0.0 ± 0.0	0.0 ± 0.0	0.794
Artery Entirety	0.0 ± 0.0	0.0 ± 0.0	0.0 ± 0.0	0.726
Ostium + Artery Entirety	0.0 ± 4.0	0.0 ± 4.0	0.0 ± 4.0	0.568
Additional Artery (%)	10/80	4/31	6/49	1.000
Infarction (%)	8/80	4/31	9/49	0.452
Left				
Ostium Morphology	0.0 ± 2.0	2.0 ± 2.0	0.0 ± 2.0	0.223
Ostium Circumference	0.0 ± 1.3	1.0 ± 2.0	0.0 ± 1.0	0.115
Ostium Entirety	0.0 ± 4.0	3.0 ± 4.0	0.0 ± 4.0	0.134
Artery Morphology	0.0 ± 0.0	0.0 ± 0.0	0.0 ± 0.0	0.688
Artery Circumference	0.0 ± 0.0	0.0 ± 0.0	0.0 ± 0.0	0.711
Artery Length	0.0 ± 0.0	0.0 ± 0.0	0.0 ± 0.0	0.607
Artery Entirety	0.0 ± 0.0	0.0 ± 0.0	0.0 ± 0.0	0.661
Ostium + Artery Entirety	0.0 ± 5.0	3.0 ± 5.0	0.0 ± 5.0	0.112
Additional Artery (%)	11/80	5/31	6/49	0.947
Infarction (%)	10/80	2/31	3/49	0.486

**Table 4:** Univariate binary logistic regression analysis with regard to the presence of AKI and various radiological renal parameters.

AKI Binary Logistic Regression	Univariate	
	Hazard Ratio (95% CI)	*p*-Value
Overall		
Ostium Entirety	1.232 (0.785–1.932)	0.364
Artery Entirety	1.056 (0.675–1.652)	0.812
Ostium + Artery Entirety	1.173 (0.750–1.835)	0.485
Additional Artery	1.086 (0.694–1.699)	0.718
Infarction	1.008 (0.642–1.584)	0.972
Right		
Ostium Morphology	1.008 (0.639–1.591)	0.972
Ostium Circumference	1.035 (0.657–1.630)	0.881
Ostium Entirety	1.054 (0.672–1.653)	0.819
Artery Morphology	1.130 (0.721–1.772)	0.593
Artery Circumference	0.920 (0.574–1.474)	0.728
Artery Length	1.063 (0.676–1.671)	0.793
Artery Entirety	1.065 (0.681–1.666)	0.782
Ostium + Artery Entirety	1.068 (0.681–1.672)	0.775
Additional Artery	1.000 (0.628–1.592)	1.000
Infarction	0.825 (0.498–1.367)	0.454
Left		
Ostium Morphology	1.321 (0.836–2.089)	0.233
Ostium Circumference	1.334 (0.845–2.105)	0.216
Ostium Entirety	1.389 (0.882–2.187)	0.157
Artery Morphology	1.018 (0.646–1.603)	0.940
Artery Circumference	0.951 (0.598–1.513)	0.833
Artery Length	1.126 (0.718–1.765)	0.606
Artery Entirety	1.035 (0.660–1.622)	0.881
Ostium + Artery Entirety	1.262 (0.806–1.977)	0.309
Additional Artery	1.073 (0.677–1.700)	0.764
Infarction	1.182 (0.736–1.899)	0.488

**Table 5:** Linear regression analysis of different perioperative creatinine levels and radiological renal parameters.

Linear Regression	Intercept (SE)	Slope (SE)	95%CI (Slope)	t-Value (Slope)	R^2^	*p*-Value (Slope)
Creatinine D-1						
Ostium Entirety—Overall	1.129 (0.074)	0.203 (0.076)	0.052–0.354	2.685	0.087	0.009
Artery Entirety—Overall	1.134 (0.072)	0.276 (0.075)	0.127–0.424	3.696	0.152	<0.001
Ostium + Artery Entirety—Overall	1.132 (0.072)	0.269 (0.074)	0.121–0.417	3.610	0.146	0.001
Additional Artery—Overall	1.128 (0.074)	0.217 (0.074)	0.070–0.364	2.933	0.102	0.004
Infarction—Overall	1.125 (0.076)	−0.145 (0.076)	−0.297–0.007	−1.905	0.046	0.061
Creatinine D0						
Ostium Entirety—Overall	1.315 (0.084)	0.164 (0.084)	−0.003–0.332	1.952	0.047	0.055
Artery Entirety—Overall	1.315 (0.084)	0.163 (0.084)	−0.005–0.330	1.936	0.046	0.057
Ostium + Artery Entirety—Overall	1.315 (0.083)	0.185 (0.084)	0.019–0.351	2.216	0.059	0.030
Additional Artery—Overall	1.315 (0.084)	0.131 (0.085)	−0.038–0.300	1.543	0.030	0.127
Infarction—Overall	1.315 (0.085)	−0.089 (0.086)	−0.259–0.082	−1.036	0.014	0.304
Creatinine D1						
Ostium Entirety—Overall	1.357 (0.089)	0.157 (0.089)	−0.021–0.335	1.756	0.039	0.083
Artery Entirety—Overall	1.357 (0.090)	0.126 (0.090)	−0.053–0.304	1.401	0.025	0.165
Ostium + Artery Entirety—Overall	1.357 (0.089)	0.162 (0.089)	−0.015–0.340	1.821	0.025	0.073
Additional Artery—Overall	1.357 (0.090)	0.118 (0.090)	−0.061–0.297	1.314	0.022	0.193
Infarction—Overall	1.359 (0.089)	−0.126 (0.090)	−0.305–0.052	−1.407	0.025	0.164
Creatinine D2						
Ostium Entirety—Overall	1.515 (0.105)	0.155 (0.106)	−0.056–0.365	1.464	0.027	0.147
Artery Entirety—Overall	1.515 (0.106)	0.162 (0.106)	−0.049–0.372	1.531	0.029	0.130
Ostium + Artery Entirety—Overall	1.515 (0.105)	0.179 (0.105)	−0.031–0.388	1.696	0.036	0.094
Additional Artery—Overall	1.515 (0.105)	0.170 (0.105)	−0.040–0.380	1.608	0.032	0.112
Infarction—Overall	1.515 (0.105)	−0.089 (0.107)	−0.301–0.124	−0.830	0.009	0.409
Creatinine D3						
Ostium Entirety—Overall	1.322 (0.117)	0.060 (0.116)	−0.173–0.294	0.519	0.005	0.606
Artery Entirety—Overall	1.321 (0.116)	0.081 (0.108)	−0.137–0.298	0.744	0.011	0.460
Ostium + Artery Entirety—Overall	1.319 (0.117)	0.084 (0.114)	−0.148–0.310	0.713	0.010	0.479
Additional Artery—Overall	1.333 (0.113)	0.207 (0.117)	−0.027–0.442	1.775	0.039	0.082
Infarction—Overall	1.324 (0.117)	−0.050 (0.135)	−0.322 –0.221	−0.370	0.003	0.713
Creatinine D4						
Ostium Entirety—Overall	1.334 (0.123)	0.057 (0.119)	−0.183–0.296	0.475	0.005	0.637
Artery Entirety—Overall	1.328 (0.121)	0.114 (0.109)	−0.105–0.333	1.051	0.023	0.299
Ostium + Artery Entirety—Overall	1.325 (0.123)	0.099 (0.117)	−0.136–0.334	0.850	0.015	0.400
Additional Artery—Overall	1.358 (0.117)	0.214 (0.120)	−0.028–0.456	1.777	0.064	0.082
Infarction—Overall	1.339 (0.117)	−0.236 (0.128)	−0.492–0.021	−0.263	0.069	0.071

**Table 6:** Univariate and multivariable cox hazard regression analysis detecting short-term mortality in dependence of different radiological renal parameters.

Cox Hazard Regression	Univariate		Multivariable	
	Hazard Ratio (95% CI)	*p*-Value	Hazard Ratio (95% CI)	*p*-Value
1-Month Mortality				
Ostium Entirety—Overall	1.340 (0.700–2.568)	0.377		
Artery Entirety—Overall	1.505 (0.919–2.465)	0.104		
Ostium + Artery Entirety—Overall	1.518 (0.838–2.750)	0.168		
Additional Artery—Overall	1.742 (1.038–2.924)	0.036	1.747 (1.024–2.979)	0.041
Infarction—Overall	1.571 (0.934–2.640)	0.088	1.563 (0.918–2.661)	0.100
6-Month Mortality				
Ostium Entirety—Overall	1.314 (0.860–2.008)	0.207		
Artery Entirety—Overall	1.246 (0.844–1.840)	0.268		
Ostium + Artery Entirety—Overall	1.339 (0.883–2.030)	0.170		
Additional Artery—Overall	1.327 (0.902–1.953)	0.151		
Infarction—Overall	1.300 (0.873–1.934)	0.196		
12-Month Mortality				
Ostium Entirety—Overall	1.314 (0.860–2.008)	0.207		
Artery Entirety—Overall	1.246 (0.844–1.840)	0.268		
Ostium + Artery Entirety—Overall	1.339 (0.883–2.030)	0.170		
Additional Artery—Overall	1.327 (0.902–1.953)	0.151		
Infarction—Overall	1.300 (0.873–1.934)	0.196		

## Data Availability

The data underlying this article will be shared upon reasonable request to the corresponding author.
